# The Prognostic Implications of Macrophages Expressing Proliferating Cell Nuclear Antigen in Breast Cancer Depend on Immune Context

**DOI:** 10.1371/journal.pone.0079114

**Published:** 2013-10-29

**Authors:** Michael J. Campbell, Denise Wolf, Rita A. Mukhtar, Vickram Tandon, Christina Yau, Alfred Au, Frederick Baehner, Laura van’t Veer, Donald Berry, Laura J. Esserman

**Affiliations:** 1 Department of Surgery, University of California, San Francisco, San Francisco, California, United States of America; 2 Department of Laboratory Medicine, University of California, San Francisco, San Francisco, California, United States of America; 3 Department of Pathology, University of California, San Francisco, San Francisco, California, United States of America; 4 Department of Biostatistics, M.D. Anderson Cancer Center, University of Texas, Houston, Texas, United States of America; 5 Agendia, Amsterdam, The Netherlands; King's College London, United Kingdom

## Abstract

Tumor associated macrophages (TAMs) are recruited from the circulation to the tumor site, and can undergo a spectrum of phenotypic changes, with two contrasting activation states described in the literature: the M1 and M2 phenotypes. We previously identified a population of TAMs that express proliferating cell nuclear antigen (PCNA) and are associated with high grade, hormone receptor negative breast cancers and poor outcomes. In the present exploratory study we again found that high PCNA^+^ TAM counts in pre-treatment tumor biopsies (102 invasive breast cancer cases from the I-SPY 1 Trial, a prospective neoadjuvant trial with serial core biopsies and gene array data) were associated with high grade, hormone receptor negativity, and decreased recurrence free survival. We explored the association of these PCNA^+^ TAMs with the expression of M1 and M2 related genes and, contrary to expectation, observed that high PCNA^+^ TAM levels were associated with more M1- than M2-related genes. An immune gene signature, derived from cytotoxic T cell and MHC Class II genes (Tc/ClassII), was developed and we found that high PCNA^+^ TAM counts, in the context of a low Tc/ClassII signature score, were associated with significantly worse recurrence free survival in all cases and in hormone receptor negative only cases. We observed similar results using a gene signature-proxy for PCNA^+^ TAMs in a larger independent set of 425 neoadjuvant-treated breast cancer cases. The results of this exploratory study indicate that high numbers of PCNA^+^ TAMs, in the absence of an anti-tumor immune microenvironment (as indicated by a low Tc/ClassII signature score), are associated with poor outcomes in breast cancer patients treated with neoadjuvant chemotherapy. This, along with the observation that PCNA^+^ TAMs were associated predominantly with M1-related genes, may provide new insights into the role of the immune microenvironment in breast cancer.

## Introduction

Solid tumors are infiltrated with leukocytes (predominately lymphocytes and macrophages) and the cross-talk between these immune cells and the cancer cells are likely to have profound effects on tumor progression. The presence of tumor-associated macrophages (TAM) represents one of the hallmarks of cancer-associated inflammation. Depending on their phenotype and the chemokines and cytokines they produce, TAMs can either facilitate tumor growth and metastasis or they can facilitate antitumor immune responses and tumor destruction [1], [2], [3]. M1 macrophages can elicit antitumor responses as a result of the secretion of pro-inflammatory cytokines such as IFN-γ, IL-12, or TNF-α [1], [2], [4]. In contrast, M2 macrophages suppress immune responses as a result of the secretion of anti-inflammatory cytokines such as IL-10, IL-13, and TGF-β and stimulate angiogenesis and tumor growth as a result of the secretion of IL-17, IL-23, vascular endothelial growth factors (VEGFs), and fibroblast growth factors (FGFs) [1], [2], [4].

Macrophages are often found in abundance in breast cancers and have been associated with poor prognosis [5], [6], [7]. We have previously reported on a subpopulation of TAMs identified by immunohistochemical staining for the expression of proliferating cell nuclear antigen (PCNA) and the macrophage marker CD68 [8]. We found that these PCNA^+^ TAMs were associated with high grade, hormone receptor negative breast cancers and poor outcomes. In a subsequent study, we reported that breast cancers in African American and Hispanic women had elevated numbers of these PCNA^+^ TAMs [9].

Since we have shown that PCNA^+^ TAMs are associated with poor outcomes in breast cancer and since M2 macrophages are associated with the production of anti-inflammatory cytokines and pro-angiogenic growth factors, one aim of the present study was to test the hypothesis that PCNA^+^ TAMs are associated with the expression of M2-related macrophage genes. In addition, due to their diverse functions and plasticity, and their potential role in either promoting tumor growth or in assisting cellular immune responses against tumors, TAMs could be markers of good or bad prognosis depending on the type of immune microenvironment they inhabit. Therefore, a second aim of this study was to explore the association of PCNA^+^ TAMs, the tumor immune microenvironment, and outcomes in women with breast cancer treated with neoadjuvant chemotherapy. 

## Materials and Methods

### Ethics Statement

The I-SPY 1 TRIAL was a collaboration of the American College of Radiology Imaging Network (ACRIN), Cancer and Leukemia Group B (CALGB, now part of the Alliance for Clinical Trials in Oncology), and the National Cancer Institute (NCI)’s Specialized Programs of Research Excellence (SPORE) [10], [11], [12]. It consisted of two protocols developed to identify markers of response to conventional neoadjuvant chemotherapy: CALGB 150007 (molecular marker component) and ACRIN 6657/CALGB 150012 (imaging component). The study was approved by nine institutional review boards responsible for the nine participating study sites: Georgetown University Hospital, Memorial Sloan Kettering Cancer Center, University of Alabama at Birmingham, University of California San Francisco, University of Chicago, University of North Carolina, University of Pennsylvania Medical Center, University of Texas Southwestern, and University of Washington. Patients signed one combined informed consent form before joining the study, which allowed them to simultaneously enroll onto the CALGB and ACRIN protocols.

### Study design and the problem of multiplicities

Our group has carried out many analyses on the I SPY-1 gene expression data set (and other expression data sets) in addition to those reported here, some related to immune function genes and others not. Therefore, due to the potential problem of multiplicities [13], this study should be viewed as exploratory. It was not designed to identify prognostic or predictive biomarkers, but rather to explore and better understand the role of macrophages and the tumor immune microenvironment in breast cancer. In Appendix S1, we outline what we had planned to do and analyses that varied from our initial plan in an attempt to address at least some of the potential silent multiplicities.

### Microarray and clinical data

**Figure 1:**
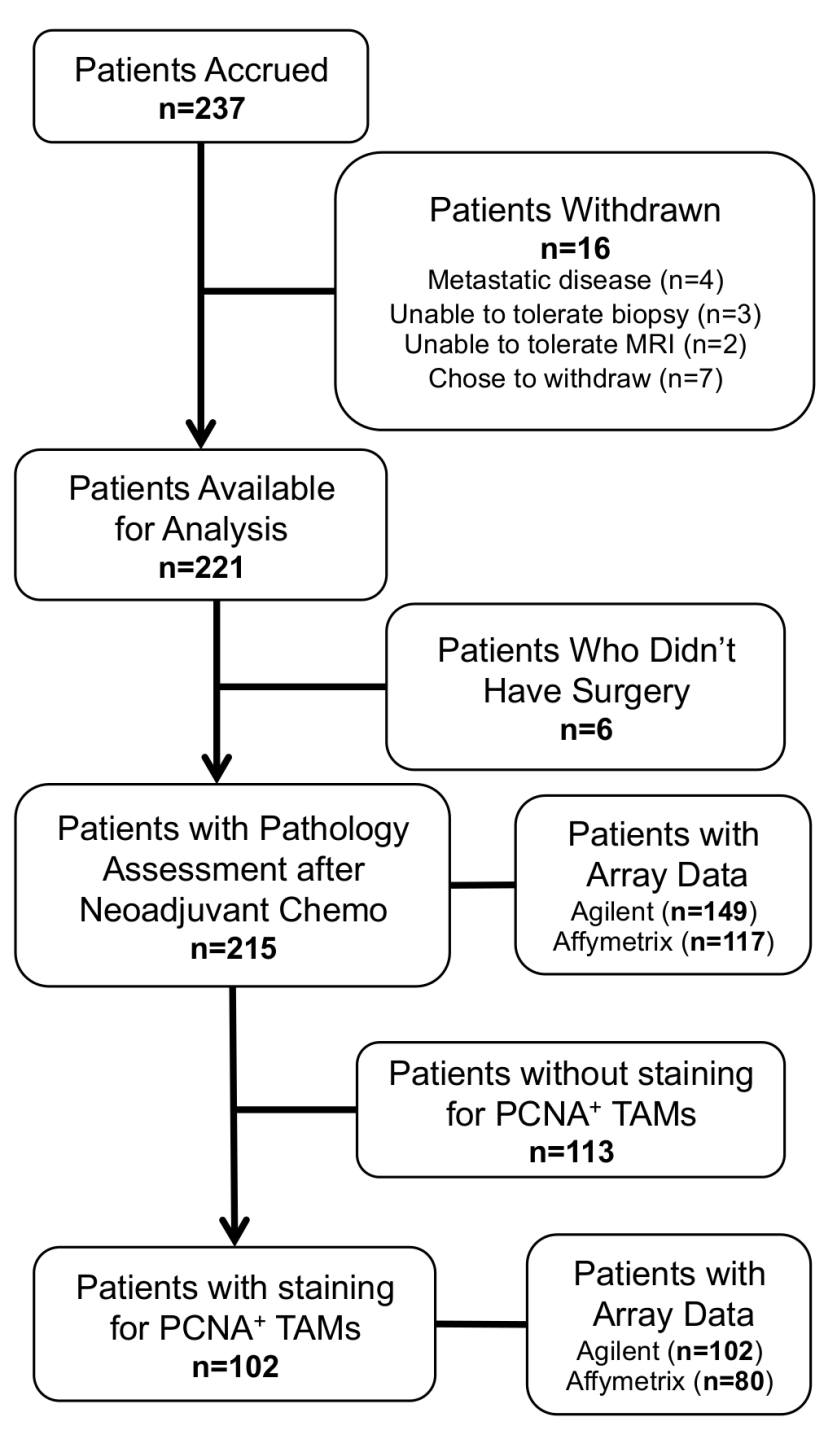
CONSORT diagram. Of the 237 patients enrolled in the I SPY-1 trial, 221 patients were evaluable, 215 had pathology results, and 102 of these were stained for PCNA^+^ TAMs. All 102 cases with IHC staining for PCNA^+^ TAMs had Agilent gene expression array data available and 80 of the 102 cases had Affymetrix expression array data available.

The microarray data used in this study was obtained from tumors of women with breast cancer treated with neoadjuvant chemotherapy on the I-SPY 1 TRIAL (2003-2006) [10], [11], [12]. Clinical data (hormone receptor status, HER2 positivity, grade, etc.) and outcomes including residual cancer burden (pCR/RCB) in the surgical sample were obtained for these patients. Details of the patient population, RCB quantification, and gene expression data have been published elsewhere [10]. Expression profiles of breast tumors biopsied prior to neoadjuvant chemotherapy were available on Agilent (149 patients) as well as Affymetrix arrays (117 patients), with an overlap of 113 patients with expression assayed on both platforms. These data are available in the GEO database under accession numbers GSE22226, GSE25055, GSE25066. Agilent array data, pre-processed by subtracting the background signal and Lowess normalizing, was used when examining M1 and M2 macrophage genes since there were more cases (102 pts) with both Agilent array data and IHC staining results (compared to Affymetrix array data and IHC data (80 pts))(see CONSORT diagram, Figure 1). For generating the Tc/ClassII signature and the gene surrogate for PCNA^+^ TAMs, RMA normalized Affymetrix array data was used to allow for subsequent testing on a larger independent data set for which expression array data was available only on the Affymetrix platform. This independent data set included 425 women with breast cancer treated with neoadjuvant chemotherapy [14]. Array data, clinical parameters, and outcomes were available on all 425 patients.

### Immunohistochemistry

We obtained an unstained tissue section from formalin fixed paraffin embedded (FFPE) core biopsies on 102 patients with Agilent 44K gene expression from the I-SPY 1 TRIAL (see Figure 1 CONSORT diagram for details). Tissue immunohistochemistry was performed on FFPE tissue sections using a standard streptavidin-biotin peroxidase method. 5 µm sections were deparaffinized in xylene and rehydrated using graded ethanol. Antigen retrieval was performed using microwave-heated 10mM citrate buffer for 10 minutes. Double staining of CD68 and PCNA was performed using dual endogenous enzyme block (Dako #3006) for 10 minutes and incubation with anti-CD68 mouse monoclonal antibody (Dako #M0876, 1:50 dilution) for 30 minutes, followed by a second incubation with anti-PCNA mouse monoclonal antibody (Dako #M0879, 1:500 dilution) overnight. For anti-CD68, DAB plus (Dako #K1395) was used as a substrate and for anti-PCNA, BCIP/NPT substrate (Dako #K0598) was used. The slides were counterstained with periodic acid Schiff reagent (American Master Tech Scientific #KTPAS) for 10 minutes at room temperature. Two pathologists independently evaluated the immunostains without knowledge of clinical outcomes or the results of previous immunostains. Slides were scanned at low power (20X) to determine three “hotspots” of positive staining. Positive staining cells (brown CD8^+^ cells with blue nuclear PCNA staining) were then counted in 3 high power fields (HPF) (100X) and the mean number was calculated [8].

### Characterization of M1- and M2-related gene expression

Genes representing M1 and M2 polarization patterns were chosen from the published literature as previously described [15]. Briefly, a gene was classified as M1-related (classical activation) if there is evidence in the literature that it can be induced in macrophages and/or monocytes following stimulation with IFN-γ and/or LPS. A gene was classified as M2-related if there is evidence in the literature that it can be induced by Th2 cytokines (IL-4 and IL-13; alternative activation), IL-10, TGF-β, glucocorticoids, or M-CSF. Genes down-regulated upon exposure to M1 polarization stimuli were also classified as M2. Genes that can be induced by both M1 and M2 stimuli were excluded from the analyses. Based on these criteria, as well as being represented on the Agilent array, 38 M1-related genes (represented by 55 probes on the array) and 29 M2-related genes (represented by 44 probes on the array) were selected for analysis (Table S1). 

### Tc/ClassII signature

The Tc/ClassII signature was derived from a literature review of cytotoxic T cell related genes and a set of MHC Class II related genes. The Tc related genes included phenotypic markers (*CD2, CD3G, CD8A*) as well as functional markers (*IFNG, TNF, GZMB, GZMH, PRF1, ZAP70*). The MHC Class II related genes included all HLA Class II genes on the Affymetrix array (*HLA-DMA, HLA-DOA, HLA DOB, HLA-DPA1, HLA-DPB1, HLA-DQA1, HLA-DQB1, HLA-DQB2, HLA-DRA, HLA-DRB1, HLA-DRB2, HLA-DRB3, HLA-DRB4, HLA-DRB5, HLA-DRB6*), the class II transactivator gene (*CIITA*), and the class II invariant chain (*CD74*).

The sum of the expression values of the Tc/ClassII probe sets was used as the signature score for each subject and the subjects were split into high- and low-score groups greater and less than the median Tc/ClassII score.

### Gene surrogate for PCNA^+^ TAMs

A gene surrogate for PCNA^+^ TAMs was developed by applying a cross-validated machine learning approach to a panel of 252 literature derived macrophage- and immune-related genes (396 probes). First, a robust voting method was used to identify genes that were highly correlated to PCNA^+^ TAMs counts (rho>0, Benjamini Hochberg (BH) adjusted p<0.005). Correlations were evaluated over patient subsets derived from 4-fold population sampling applied 200 times (800 random sets of 60 of the 80 samples with TAMs and AFFY expression data), with each probe receiving a ‘vote’ every time its correlation coefficient achieved significance (BH p<0.005). The 6 probes with the most ‘votes’ were used as potential features in the predictive model. To derive a signature with optimal weights for these candidate features, we applied non-negative least squares regression against the PCNA^+^ TAMs counts, using the function nnls.R in Bioconductor[16]. ROC analysis of this signature against High (>24 per HPF) versus Low (<=24 per HPF) PCNA^+^ TAM counts was used to derive an optimal Youden scoring threshold *Y_t_* (via functions roc.R and cords.R). A sample with PCNA^+^ TAMs surrogate score *S_TAM_* greater than *Y_t_* was predicted to be in the high PCNA^+^ TAM class. Otherwise, a sample was predicted to be in the low PCNA^+^ TAM class. To estimate the performance of the PCNA^+^ TAMs surrogate, we performed 4-fold cross-validation 200 times. This entailed re-fitting the regression on each of the 800 training sets (60 samples each) and evaluating the AUC on each associated test set (20 samples each). Performance was summarized as the mean AUC over all cross-validations. Randomizations into folds were balanced throughout to maintain similar proportions of high and low PCNA^+^ TAMs samples, and HR- positive and –negative tumors. 

### Statistical analyses

Fisher’s exact test or Student’s t-test were used to determine associations between PCNA^+^ TAMs and various clinicopathological parameters. For both the I SPY-1 data set and the independent, confirmatory data set, Kaplan-Meier curves were used to show recurrence free survival (RFS) for the various subgroups, defined as patients with low or high PCNA^+^ TAMs (or surrogate), low or high Tc/Class II score (defined above), as well as patients with high PCNA^+^ TAMs (or surrogate) AND low Tc/Class II score versus all others. Time-to-event distributions were compared using log rank tests and univariate and multivariate proportional hazards modeling performed with and without adjustments for HR status and tumor grade. M1- and M2-related genes that were differentially expressed in high and low PCNA^+^ TAMs subgroups were identified using a two-tailed t-test with a significance threshold of p<0.05, and visualized on volcano plots of the difference of means (x-axis) plotted against p values (y-axis). Pearson’s product moment correlation coefficient was also used to estimate the association between M1- or M2-related gene expression and (continuous) PCNA^+^ TAM counts. Fisher’s exact test was used to determine whether a greater number of M2 than M1 genes were differentially expressed in high and low PCNA^+^ TAMs subgroups. Analyses were conducted using Bioconductor R [16]. Unless otherwise specified, all reported p values are uncorrected for multiple comparisons (where indicated, p values were corrected for multiple comparisons using the Benjamini-Hochberg (BH) method).

## Results

### PCNA^+^ TAMs associate with high grade, hormone receptor negativity, and poor outcomes

**Figure 2:**
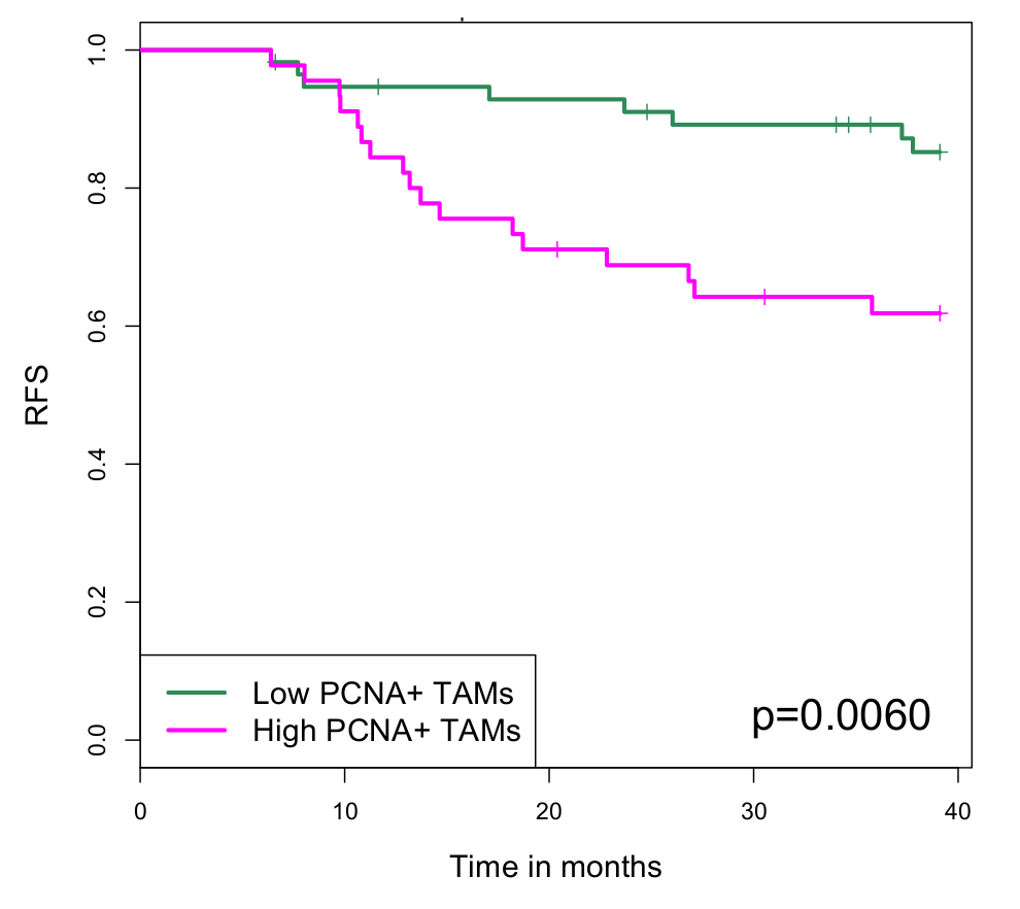
Kaplan-Meier plot of recurrence free survival (RFS) in breast cancer patients from the I SPY-1 cohort. Patients were stratified by high (n=45) and low (n=57) PCNA^+^ TAMs. The difference between groups was significant using the log rank test (p=0.006).

One hundred and two breast cancer cases from I-SPY 1 were stained with anti-PCNA and anti-CD68 antibodies and the double positive PCNA^+^ TAMs enumerated as described above. The mean number of PCNA^+^ TAMs per high-power field (23.6 +/- 1.5; mean +/- SEM) was used to dichotomize cases into high (>24) or low (<=24) groups. As we have previously reported, PCNA^+^ TAMs were significantly associated with high grade, hormone receptor (HR) negative breast cancers (Table 1). There was no association with age or number of positive nodes. Intrinsic subtypes and wound healing gene signatures were determined from expression array data as published [10] and high PCNA^+^ TAMs were found to be associated with Basal and Her2 subtypes as well as an activated wound healing signature (Table 1). High PCNA^+^ TAMs were also significantly associated with high staining for Ki67 by IHC, but not with staining for EGFR or CyclinD1. Finally, high PCNA^+^ TAMs were associated with decreased recurrence free survival (p=0.006; Figure 2).

**Table 1:** Relationship between clinical factors and PCNA^+^ TAMs in breast cancer.

		**PCNA^+^ TAMs**	**PCNA^+^ TAMs**	
**Characteristic**		**Low (<=24)**	**High (>24)**	**p-value**
Age at diagnosis (mean)		48.2	47.4	0.6514^a^
Number of positive nodes (mean)		2.1	2.5	0.6076 ^a^
HR positive		43	16	**4.65E-05^b^**
HR negative		14	29	
Grade 1,2		33	13	**0.0049 ^b^**
Grade 3		24	32	
Wound healing signature	quiescent	14	3	**0.0175 ^b^**
	active	43	42	
Intrinsic subtypes	Basal	16	22	
	Luminal A	27	3	
	Luminal B	10	11	
	HER2	3	8	
	Normal	1	2	
	Basal vs. Luminal A&B			**0.0040^b^**
	HER2 vs. Luminal A&B			**0.0054 ^b^**
Ki67 IHC - high				**0.0006 ^b^**
Ki67 IHC - intermediate				
Ki67 IHC - low				
Cyclin D1 IHC - neg				0.0739 ^b^
Cyclin D1 IHC - pos				
EGFR IHC - neg				0.1692 ^b^
EGFR IHC - pos				

^a^ Student’s t-test

^b^ Fisher’s exact test

Of the 102 cases stained for PCNA^+^ TAMs, residual cancer burden (RCB) data following neoadjuvant chemotherapy and surgical excision was available for 90 cases. Sixty percent of the patients who achieved a pathological complete response (pCR; RCB 0) or had minimal residual disease (RCB 1) fell into the high PCNA^+^ TAMs group, as did 58% of the RCB 3 cases. There was no difference in recurrence free survival in the RCB 0/1 cases with high PCNA^+^ TAMs versus low PCNA^+^ TAMs, whereas there was a significant reduction in RFS in the RCB 3 cases with high PCNA^+^TAMs compared to those with low PCNA^+^ TAMs (p = 0.0398). Thus, although high PCNA^+^ TAMs can be found in patients with good or poor responses to chemotherapy, they were associated with negative prognostic value only in those patients with significant residual disease.

### PCNA^+^ TAMs associate with more M1 than M2 macrophage genes

**Figure 3:**
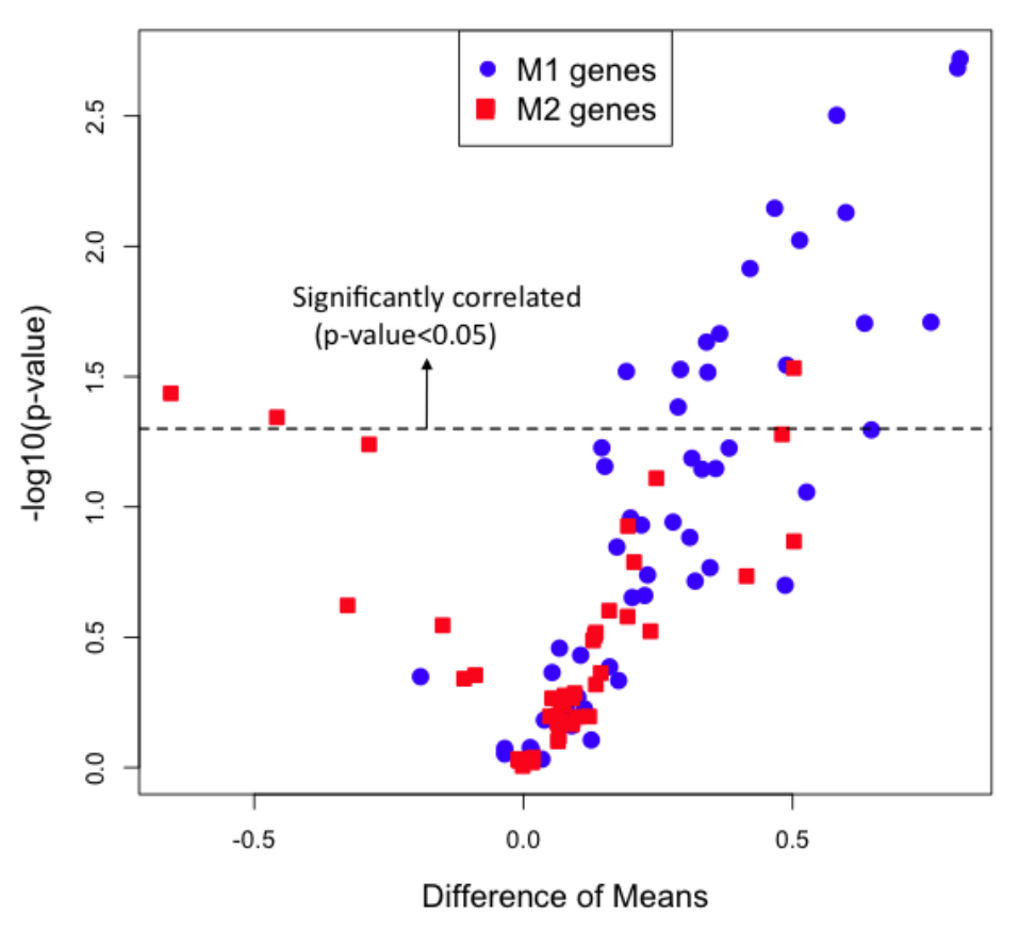
Differential expression of M1- and M2-related genes. Volcano plot of M1- and M2-related probe sets differentially expressed between patients with high PCNA^+^ TAMs and low PCNA^+^ TAMs. The x-axis corresponds to the difference of the mean expression values for a given probe (high PCNA^+^TAMs minus low PCNA^+^ TAMs), and the y-axis corresponds to the p value (Student’s t test). The horizontal dashed line indicates the p=0.05 cutpoint for significance. These results were not corrected for multiple testing.

Since M2 macrophages tend to be pro-tumorigenic and PCNA^+^ TAMs are associated with poor outcomes, we hypothesized that PCNA^+^ TAMs would be associated with the expression of M2-related genes. To test this hypothesis, we used two-tailed t-tests to compare the expression of a panel of pre-selected macrophage-related genes (representing both M1 and M2 polarized macrophages) in the high versus low PCNA^+^ TAMs groups and found predominantly M1-related genes were differentially overexpressed in cases with high PCNA^+^ TAMs (Figure 3). Of the 38 M1-related genes (represented by 55 probes on the Agilent array), 15 genes (39%; represented by 16 probes) were overexpressed in tumors with high PCNA^+^ TAMs compared to low PCNA^+^ TAMs (uncorrected p<0.05; Figure 3 and Table S1). In contrast, of the 29 M2-related genes (represented by 44 probes), only 2 genes (7%; represented by 3 probes) were differentially expressed. Thus there were significantly more M1-related than M2-related genes differentially expressed in high versus low PCNA+ TAMs subgroups (Fisher’s exact test: p=0.0038). One of the M2-related genes, CCL18 showed higher expression in the high PCNA^+^ TAMs cases while the other, CD36 showed higher expression in the low PCNA^+^ TAMs cases (Table S1). 

Among the M1-related genes up-regulated in the high PCNA^+^ TAMs group were the interferon-γ (IFN-γ) inducible chemokines *CXCL10* (p=0.009), *CXCL11* (p=0.002), and *CCL4* (p=0.003) as well as IFN regulatory factor 1 (IRF1; p=0.026) and guanylate binding proteins *GBP4* (p=0.019) and *GBP5* (p=0.002) which have been implicated in IFN signaling. Several of the up-regulated M1-type genes are associated with Toll-like receptor signaling and innate immune responses: *TLR2* (p=0.008), *TNF* (p=0.021), *IL1B* (p= 0.041), *IL32* (p=0.021), and *NOS2A* (p=0.033). Other up-regulated M1-type genes were the costimulatory molecule *CD86* (p=0.012), the Fc gamma receptors *FCGR1* (p=0.027) and *FCGR2A* (p=0.029), and the adhesion molecule *ICAM1* (p=0.007). 

Since the M1/M2 gene lists were hand-selected for biological significance, the p-values listed above are not corrected for multiple testing. With a more stringent significance criteria applied using Benjamini-Hochberg multi-test correction (BH p<0.05), 7 of the 38 M1-related genes and no M2-related genes were found to be differentially expressed. These additional calculations confirm that contrary to expectation, more M1- than M2-related genes are differentially regulated in high PCNA+TAMs cases (Fisher’s exact test; p=0.0163). 

We also examined the relationship between M1- and M2-related genes and various clinical parameters. Similar to what we observed comparing high vs. low PCNA+ TAMs cases, more M1- than M2-related genes were differentially up-regulated in HR negative tumors vs. HR positive tumors, in high grade vs. low grade tumors, and in patients with no or minimal residual cancer (RCB 0/1) vs. high residual cancer burden (RCB 3). Interestingly, comparing patients whose cancer recurred vs. those who remained recurrence free there were 9 M1 genes and 8 M2 genes differentially up-regulated in the recurrence free cases (Table S2).

### High PCNA^+^ TAMs associate with poor outcome in a suppressed tumor immune microenvironment

Since TAMs can either promote tumor growth or assist cellular immune responses against tumors, we hypothesized that their prognostic significance depends on their context within the tumor immune microenvironment and that high numbers of PCNA+ TAMs in association with a suppressed tumor immune microenvironment would predict poor outcomes.

**Figure 4:**
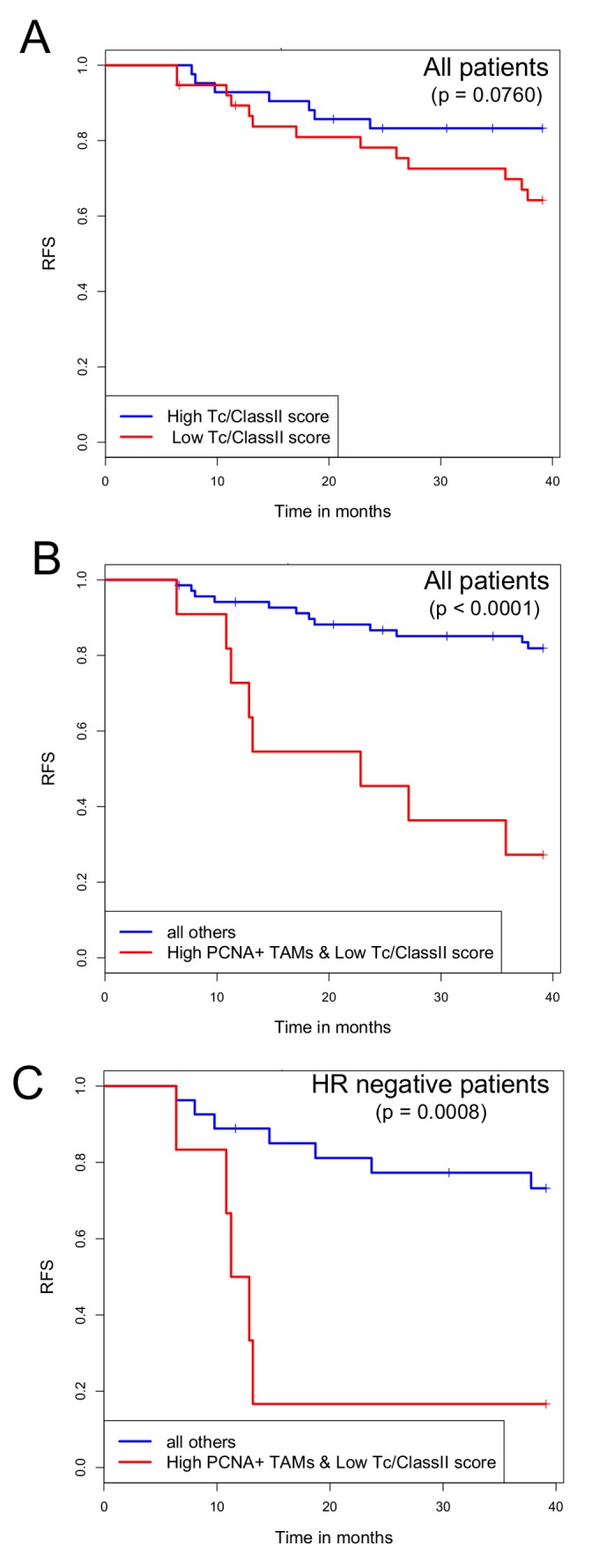
Kaplan-Meier plots of recurrence free survival in breast cancer patients from the I SPY-1 cohort predicted by the Tc/ClassII immune signature score, alone and in conjunction with PCNA^+^ TAM counts. (A) Kaplan-Meier curves for recurrence free survival (RFS) were stratified by the median Tc/ClassII signature score into low (n=38) or high (n=42) subsets. (B) Kaplan-Meier curves for RFS comparing patients with high PCNA^+^ TAMs and a low Tc/ClassII score (n=11) to all other patients (n=69). (C) Kaplan-Meier curves for RFS in hormone receptor negative cases only; high PCNA^+^ TAMs and a low Tc/ClassII score (n=7) to all other HR negative cases (n=26). Differences between groups were assessed using the log rank test, with p values indicated in each plot.

To test this hypothesis, we selected a panel of cytotoxic T cell related genes and a panel of MHC ClassII related genes to generate a Tc/ClassII signature (see Methods). A low Tc/ClassII signature score would be indicative of a suppressed tumor immune microenvironment. Using the median Tc/ClassII signature score as a cut-point, we found that on its own, this immune signature did not separate cases with good versus poor outcomes over the 3 years of follow-up in the study (Figure 4A). However, using the Tc/ClassII signature along with PCNA^+^ TAM counts we found that patients with a low Tc/ClassII score and high PCNA^+^ TAMs had significantly worse outcomes (Figure 4B, log-rank p=1e-05). Since patients with hormone receptor negative tumors tend to have poor outcomes, we looked just within this group and also found that a low Tc/ClassII score with high PCNA^+^ TAMs was associated with significantly shorter RFS (Figure 4C, log-rank p=0.0008).

In univariate analyses, high PCNA^+^ TAMs moderately associate with poor outcome (p=0.0223), whereas Tc/Class II score does not significantly associate with RFS (Table 2). In multivariate analyses, high PCNA^+^ TAMs become much more significantly associated with poor outcome (p=0.0024), as does a low Tc/ClassII score (p=0.0086), further indicating that it is the combination of high PCNA^+^ TAMs and a low Tc/ClassII score that predicts recurrence (p=7.7e-06) (Table 3). These results are maintained after models are adjusted for HR status and grade (Tables 2 and 3), reinforcing that neither TAMs nor the immune microenvironment as reflected in the Tc/ClassII score are merely proxies for known risk factors like HR negativity and high grade.

**Table 2:** Univariate and multivariate analysis of PCNA^+^ TAMs, Tc/ClassII signature score, and breast cancer outcomes.

**Factor**	**Univariate model**		**Multivariate model**		**Multivariate model adjusted for HR status and grade**	
	HR (95% CI)	p-value^a^	HR (95% CI)	p-value^a^	HR (95% CI)	p-value^a^
High PCNA+ TAMs	2.93 (1.17-7.34)	**0.0223**	4.44 (1.69-11.64)	**0.0024**	3.35 (1.27-8.89)	**0.0033**
Low Tc/ClassII Score	2.25 (0.90-5.64)	0.0842	3.62 (1.39-9.48)	**0.0086**	4.29 (1.62-11.34)	**0.0150**

^a^ Wald

**Table 3:** The combination of high PCNA^+^ TAMs and a low Tc/ClassII score predicts recurrence even after adjusting for HR status and grade.

**Comparison**		**Unadjusted model**		**Adjusted for HR status and grade**	
	N	HR (95% CI)	p-value^a^	HR (95% CI)	p-value^a^
High PCNA+ TAMs & Low Tc/ClassII score vs. all others	80	6.14 (2.48-15.16)	**7.655E-06**	5.77 (2.32-14.32)	**2.161E-05**

^a^ Log rank

### A similar pattern is observed in an independent data set

Validating these results in a larger data set would require an independent cohort of breast cancer patients treated with neoadjuvant anthracycline based chemotherapy with IHC staining results for PCNA^+^ TAMs. Since no such cohort was available, we performed a two-step analysis to investigate whether our results might apply to other populations – specifically, to an independent cohort of 425 patients with who received neoadjuvant chemotherapy and have associated expression data but not IHC staining for TAMs [14]. 

We first developed a gene surrogate for PCNA^+^ TAMs using the I-SPY 1 expression array data for a pre-selected group of macrophage-associated genes and the IHC staining for PCNA^+^ TAMs as described in Methods. Briefly, five genes that demonstrated significant correlation (BH p<0.005) with PCNA^+^ TAMs (*CCL8, CCR1, CXCL10, CXCL11, and LAMP3*) were selected by a machine learning voting method over 800 random samplings of the data. A surrogate for PCNA^+^ TAMs was derived by performing a non-negative least squares regression of these genes against the PCNA^+^ TAMs scores, yielding a four-gene signature – *CXCL10, CXCL11, CCL8*, and *LAMP3* (probes 204533_at, 210163_at, 214038_at, 211122_s_at, and 205569_at) - with optimal weights 0.648, 5.264, 3.282, 7.629, and 4.154. ROC analysis of this signature against high (>24 per HPF) versus low (<=24 per HPF) PCNA^+^ TAM counts yielded an optimal Youden scoring threshold of 5.56. Thus, samples with weighted gene expression scores (probes * weights) > 5.56 were predicted to be in the high PCNA^+^ TAM class. A 4-fold cross-validation analysis repeated 200 times produced a performance estimate of mean (AUC)=0.736 for the PCNA^+^ TAMs expression surrogate. 

**Figure 5:**
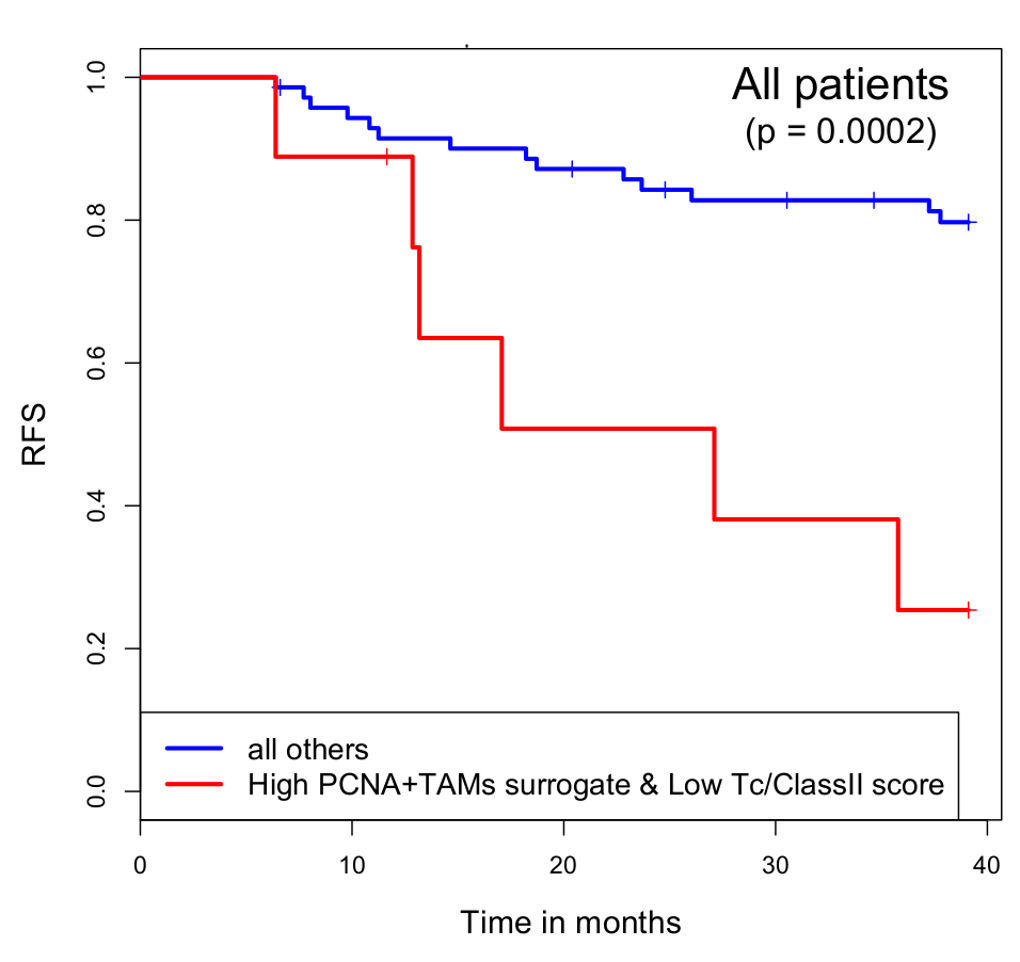
A high PCNA^+^TAMs-surrogate score, in association with a low Tc/ClassII score, is associated with poor outcomes in the I-SPY 1 patient cohort. Kaplan-Meier curves for RFS comparing patients with a high PCNA^+^ TAMs surrogate score and a low Tc/ClassII score (n=9) to all other patients (n=71). The difference between groups was significant using the log rank test (p=0.0002).

**Figure 6:**
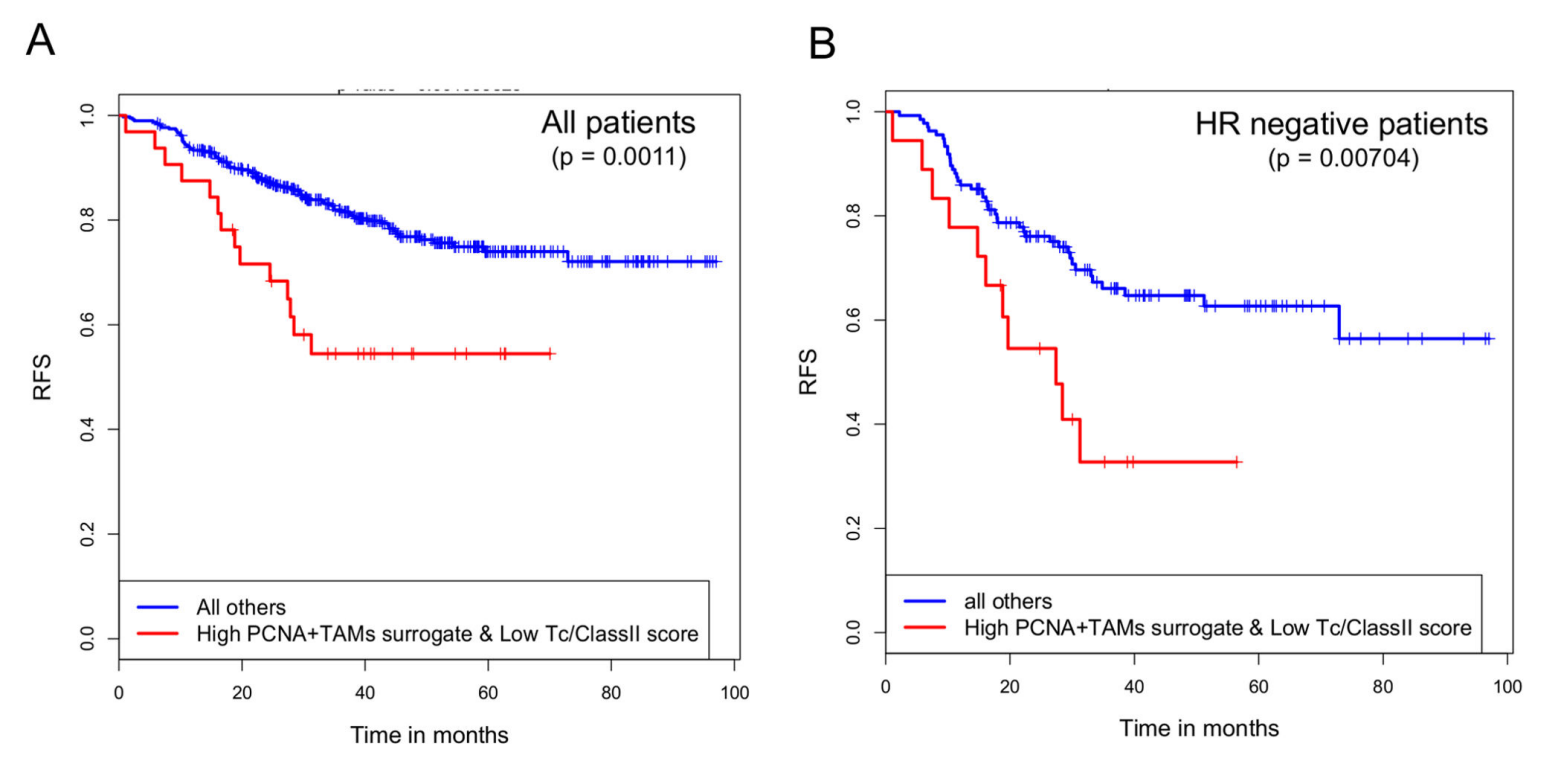
Kaplan-Meier plots of recurrence free survival in breast cancer patients from the validation set predicted by the Tc/ClassII immune signature score in conjunction with the PCNA^+^ TAMs surrogate score. (A) Kaplan-Meier curves for RFS comparing patients with a high PCNA^+^ TAMs surrogate score and a low Tc/ClassII score (n=59) to all other patients (n=366). (B) Kaplan-Meier curves for RFS in hormone receptor negative cases only; high PCNA^+^ TAMs surrogate score and a low Tc/ClassII score (n=30) to all other HR negative cases (n=123). Differences between groups were assessed using the log rank test, with p values indicated in each plot.

As shown in Figure 5, when we re-interrogated the I-SPY 1 data using the PCNA^+^ TAMs surrogate, we found that a high PCNA^+^ TAMs surrogate score, in association with a low Tc/ClassII score, predicts poor recurrence free survival in the I-SPY 1 data, similar to the results shown in Figure 4B using IHC staining for PCNA^+^ TAMs. Applying these two signatures to the larger independent cohort of 425 patients, we observed significantly worse recurrence free survival in patients with a low Tc/ClassII score and high PCNA^+^TAMs surrogate score (Figure 6A). This also held true when only hormone receptor negative cases were examined (Figure 6B). Though these results are not in a strict sense a validation of our observation from I-SPY that high numbers of PCNA^+^ TAMs in the context of suppressed antitumor immune microenvironment predict poor outcomes, they are supportive of this theme. 

## Discussion

In agreement with our previously published work [8], [9] we demonstrated herein that a subset of tumor associated macrophages, those staining positively for proliferating cell nuclear antigen (PCNA) as well as CD68 (PCNA^+^ TAMs), were associated with high grade, hormone receptor negative breast cancers with poor outcomes. CD68 expression has been reported on some granulocytes, myeloid precursors, endothelial cells and fibroblasts [17], [18]. However this is somewhat antibody dependent and the anti-CD68 antibody used in our study (clone PG-M1; Dako M0876) detects a fixative-resistant epitope on a macrophage-restricted form of the CD68 antigen, which is not detected on granulocytes, myeloid precursors, endothelial cells, or fibroblasts [17], [18].

Monocytes are recruited to tumors by chemokines produced by the tumor cells and/or stromal cells. Once within the tumor microenvironment, their differentiation is influenced by signals they receive from the tumor cells and the surrounding stroma, which can lead to either an M1 or M2 polarization of the TAMs. It has been suggested that TAMs are polarized towards an M2 phenotype, promoting tumor growth and metastasis [1], [2]. However, recent studies indicate that there are distinct TAM subpopulations in mammary tumors that do not fit neatly into a M2 phenotype. Egeblad et al. [19], identified three distinct TAM populations in the *MMTV-PyMT* mouse model of mammary carcinoma. Movahedi et al. [20], used MHC class II expression to discriminate two TAM subpopulations in the TS/A and 4T1 mouse mammary carcinoma models. In these mouse models the MHC class II high TAMs were associated with more M1-type genes such as *NOS2*, *PTGS2* (Cox2), *IL1B*, *IL6*, and *IL12B*, whereas M2-related genes such as *ARG1*, *CD163*, *STAB1*, and *MRC1* were expressed at higher levels in the MHC class II low TAMs. These TAM subsets also showed distinct chemokine expression profiles. Chemokine genes involved in lymphocyte attraction, such as *CCL5*, *CCL17*, *CCL22*, *CXCL1*, *CXCL9*, *CXCL10*, and *CXCL11* were up-regulated in the MHC class II high M1-like TAMs. In contrast, chemokines involved in monocyte/macrophage attraction, such as *CCL2*, *CCL3*, *CCL4*, *CCL6*, *CCL7*, *CCL9*, and *CCL12* were significantly higher in the MHC class II low M2-like TAMs. These experiments suggest a more complex phenotypic partitioning of TAMs than the simple M1/M2 dichotomy.

Using expression array data in combination with immunohistochemical staining, in the present study we found that tumor samples with high PCNA^+^ TAMs demonstrated higher expression of several M1-related genes, but not M2-type genes. This result was not what we had expected based on the M1/M2 paradigm that would predict these TAMs to be in the M2 state, but is not without precedent as described above. Many of these M1 genes are associated with IFN signaling, TLR signaling, and innate immune responses. Similar to the findings by Movahedi et al. [20], with MHC class II high TAMs, we observed increased expression of the M1-type genes *NOS2* and *IL1B* as well as the lymphocyte chemoattractants *CXCL10* and *CXCL11* associated with high PCNA^+^ TAMs. We also found that high numbers of PCNA^+^ TAMs were associated with higher expression of MHC class II genes (data not shown), suggesting that these PCNA^+^ TAMs may be similar to the MHC class II high M1-like TAMs observed in the TS/A and 4T1 mouse mammary carcinoma models. 

Tumor infiltrating T lymphocytes in breast cancer are a heterogeneous population of cells. Although in many reports, cytotoxic CD8^+^ T cells (Tc) are the predominant T cell present [21], [22] [23], no definitive conclusion has been drawn regarding the correlation between tumor infiltrating Tc and outcomes in breast cancer. One recent report associates infiltration of CD8^+^ T cells in human breast cancer with a good prognosis [24]. Interestingly, this group found that CD8^+^ T cells were correlated with higher tumor grade, hormone receptor negativity, and a basal phenotype, similar to our results with PCNA^+^ TAMs. In a recent report by DeNardo et al. [25], breast cancer patients with high CD8 counts also had better prognosis than patients with low CD8 counts. In addition, using expression data they found that patients with low CD68 and high CD8 expression had a better prognosis than patients with high CD68 and low CD8 expression [25]. In contrast, Mahmoud et al. reported that CD68 had no prognostic impact in subsets with low or high CD8 counts [24]. We investigated CD68 and CD8 expression in both the I-SPY 1 and the validation data sets. Using the median expression values of CD68 and CD8 as cut-points, there was no significant difference in RFS between highCD68/lowCD8 and lowCD68/highCD8 cases in either data set (data not shown). The same results were found when examining just the HR-negative subset of patients.

To further evaluate T cell function and their interaction with PCNA^+^ TAMs, we developed a gene signature comprised of several Tc related genes and MHC class II related genes. The Tc genes included phenotypic markers (*CD2*, *CD3G*, and *CD8A*) and functional markers such as the cytokines IFN-γ and TNF-α as well as granzymes and perforin, which are involved in the cytotoxic killing of target cells. The presence of high numbers of PCNA^+^ TAMs (or a high PCNA+TAM gene surrogate score) and a low Tc/Class II signature score predicted an extremely poor recurrence free survival in both the I-SPY 1 data set (n=80) and a larger independent set (n=425). Since high PCNA^+^ TAM counts are associated with hormone receptor negative (HR-neg) breast cancers and since these HR-neg cancers tend to have worse outcomes, we also examined the Tc/Class II signature and PCNA^+^ TAMs in HR-neg cases only. In both the I-SPY 1 data set and the larger independent data set, recurrence free survival of HR-neg cases with high PCNA^+^ TAM counts (or a high PCNA+TAM gene surrogate score) and a low Tc/Class II signature score was significantly worse than other groups. 

Given the significant heterogeneity of breast tumors, it should be noted that the I-SPY 1 trial is a study of mostly patients who presented with large palpable masses, and largely (91%) comprised of patients with high-risk biology, as measured by the 70-gene signature [26]. The independent data set is also largely comprised of higher risk tumors [14]. In these patients, poor immune function appears to be associated with a worse outcome and with the inability to mount an effective response to chemotherapy [27].

A limitation of this study is that since the expression array data was derived from a heterogeneous mixture of tumor cells and stromal cells, one does not know which cells are expressing which genes. Although we found an association of PCNA^+^ TAMs with M1-type but not M2-type gene expression, these genes could be from the TAMs, tumor cells, or other cells. Thus, from this data we cannot say whether PCNA^+^ TAMs are M1 or M2 cells, but only that they are more associated with an M1-type tumor immune microenvironment. We are currently evaluating M1 and M2 markers on TAMs in breast cancer tissue sections using multi-color immunohistochemistry and multi-spectral imaging tools to address this question. Another limitation of this study is that we did not have IHC staining for PCNA+ TAMs in the independent set of 425 patients, and thus needed to develop and use a gene expression surrogate. As gene expression is noisy and the I-SPY 1 population size with IHC staining for TAMs and Affymetrix gene expression data is relatively small (n=80), this surrogate may not be an accurate representation of TAM counts in other patient populations. 

## Conclusions

It must be reiterated that due to the insidious nature of multiplicities [13], this should be considered an exploratory study, albeit one that raises some interesting points for further exploration. The results of this exploratory study indicate that high numbers of PCNA^+^ TAMs, particularly in the absence of an anti-tumor immune microenvironment (as indicated by a low Tc/ClassII signature score), are associated with poor outcomes in breast cancer patients treated with neoadjuvant chemotherapy. This, along with the observation that PCNA^+^ TAMs were associated predominantly with M1-related genes, may provide new insights into the role of the immune microenvironment in breast cancer. Although initial results of immunotherapeutic strategies in breast cancer have been disappointing, the results of this study encourage further research along these lines. The challenge ahead will be to dissect the pro- and anti-tumor aspects of the breast cancer immune microenvironment with the aim of developing strategies for optimally re-educating TAMs, along with infiltrating T cells, towards an antitumor response.

## Supporting Information

Appendix S1 **Supplemental materials and methods.**
(DOCX)

Table S1 **M1- and M2-related gene expression in breast cancers with high vs. low PCNA^+^ TAMs.**
(DOCX)

Table S2 **Association of M1- and M2-related differential gene expression with clinical parameters in breast cancer.**
(DOCX)
