# Supplementary material for: The Prognostic Implications of Macrophages Expressing Proliferating Cell Nuclear Antigen in Breast Cancer Depend on Immune Context
Source: PLoS One. 2013 Oct 29;8(10):e79114. doi: 10.1371/journal.pone.0079114 (PMC3812150; doi:10.1371/journal.pone.0079114)
Supplement: Appendix S1 — Supplemental materials and methods. (DOCX) [file pone.0079114.s001.docx]

**Supplementary Materials and Methods**

***Study design and the problem of multiplicities***

For this study we planned to stain breast cancer tissues, from patients on the I SPY-1 trial, for infiltrating PCNA^+^ TAMs and to evaluate associations of PCNA^+^ TAMs with clinical parameters (age at diagnosis, number of positive nodes, hormone receptor status, grade, intrinsic subtypes). Samples were to be dichotomized into high and low PCNA^+^ TAMs using a cut-point of 5 PCNA^+^ TAMs per high power field as we had previously published [[8](#_ENREF_8)]. However, in this study overall PCNA^+^ TAM counts were much higher than our previous study and there were only three cases with counts less than 5. Thus, we changed the cut-point for high vs. low PCNA^+^ TAMs to be equal to the mean value for this study population. Student’s t-test (age, nodes) and Fisher’s exact test (HR status, grade, subtypes) were to be used for statistical analyses. There were some associations with PCNA^+^ TAMs that were not initially planned, but were subsequently examined: wound healing signature, Ki67 staining, cyclin D1 staining, and EGFR staining. We also proposed to evaluate the association of PCNA^+^ TAMs and outcomes (recurrence free survival) using Kaplan-Meier analyses (in all patients, as well as in only the HR-negative subset of patients) and in a multivariate analysis adjusting for HR status and grade since in previous studies we had found an association of PCNA^+^ TAMs with HR-negativity and high grade.

Since TAMs have been suggested to be mainly of the M2 phenotype [[1](#_ENREF_1),[2](#_ENREF_2)], we planned to evaluate the association of PCNA^+^ TAMs with M1 and M2 gene expression, using a predefined panel of M1 and M2 related genes. We would compare the expression level of each gene in tumors with high vs. low PCNA^+^ TAMs using Student’s t-test (not adjusting for multiple comparisons). During the course of the study, we included an analysis correcting for multiple comparisons using the Benjamini-Hochberg method.

Due to their diverse functions and plasticity, TAMs could be markers of good or bad prognosis depending on the type of immune microenvironment they inhabit. Initial plans were to use immunohistochemical (IHC) stains on tissue sections to identify other infiltrating leukocytes (CD8+ cytotoxic T cells, CD4+ helper T cells, and CD4+/FOXP3+ regulatory T cells). However, due to the limited supply of tissue sections available from the I SPY-1 study, we chose to examine the tumor immune microenvironment by using the available gene expression data. We focused on developing a gene expression signature derived from a predefined panel of genes associated with cytotoxic T cell responses and a predefined panel of major histocompatibility complex (MHC) class II genes. The expression values of these genes would be summed and we planned to use a median cut-point to define a high Tc/ClassII gene signature score from a low score and proposed that a low score would be indicative of a suppressed immune microenvironment. We then planned to examine the association of PCNA^+^ TAMs, the Tc/ClassII signature, and breast cancer outcomes using Kaplan-Meier analyses (in all patients, as well as in only the HR-negative subset of patients) and in a multivariate analysis adjusting for HR status and grade.

Finally, we had planned a confirmatory analysis using an independent data set of patients with breast cancer, treated with neoadjuvant chemotherapy, for which gene expression data was available. Since we would not have IHC staining data for PCNA^+^ TAMs on this data set, we planned to develop a gene surrogate for PCNA^+^ TAMs. Originally, we had planned to use the data set published by Hess et al [[14](#_ENREF_14)], but during the course of our study a different, larger data set became publicly available [[15](#_ENREF_15)] and we chose to use this larger data set. The PCNA^+^ TAMs gene surrogate was to be developed using a pre-specified panel of macrophage-related genes, selecting three genes that had the highest correlation with PCNA^+^ TAM counts, and summing their expression to yield a PCNA^+^ TAMs gene surrogate score. Although this method resulted in a gene surrogate for PCNA^+^ TAMs (data not shown), to be more robust we chose to apply a cross-validated machine learning approach as described below. Once a PCNA^+^ TAM gene surrogate was developed, we planned to use it along with the Tc/ClassII signature score in a Kaplan-Meier analysis of recurrence-free survival in both the I SPY-1 and the confirmatory data sets (in all patients and in HR-negative patients only).
