# Supplementary material for: The Prognostic Implications of Macrophages Expressing Proliferating Cell Nuclear Antigen in Breast Cancer Depend on Immune Context
Source: PLoS One. 2013 Oct 29;8(10):e79114. doi: 10.1371/journal.pone.0079114 (PMC3812150; doi:10.1371/journal.pone.0079114)
Supplement: Table S1 — M1- and M2-related gene expression in breast cancers with high vs. low PCNA+ TAMs. (DOCX) [file pone.0079114.s002.docx]

**Table S1. M1- and M2-related gene expression in breast cancers with high vs low PCNA^+^ TAMs.**

**M1 GENES**

| Gene name | Symbol | Probe set ID | difference^a^ | p value^b^ |
| --- | --- | --- | --- | --- |
| Apolipoprotein L3 | APOL3 | AGI_HUM1_OLIGO_A_23_P29237 | 0.3820 | 0.0521 |
|  | APOL3 | AGI_HUM1_OLIGO_A_24_P416997 | 0.1601 | 0.3988 |
| Complement component 3a receptor 1 | C3AR1 | AGI_HUM1_OLIGO_A_23_P2431 | 0.0802 | 0.5946 |
| Guanylate-binding protein 1, IFN-inducible | GBP1 | AGI_HUM1_OLIGO_A_23_P62890 | 0.5261 | 0.0820 |
| Guanylate-binding protein 2, IFN-inducible | GBP2 | AGI_HUM1_OLIGO_A_23_P85693 | 0.1135 | 0.5839 |
| Guanylate-binding protein 3 | GBP3 | AGI_HUM1_OLIGO_A_23_P51487 | 0.1768 | 0.4589 |
|  | GBP3 | AGI_HUM1_OLIGO_A_24_P370702 | 0.3466 | 0.1632 |
| Guanylate-binding protein 4 | GBP4 | AGI_HUM1_OLIGO_A_23_P103496 | 0.6338 | **0.0194** |
|  | GBP4 | AGI_HUM1_OLIGO_A_24_P45446 | 0.2306 | 0.1742 |
| Guanylate-binding protein 5 | GBP5 | AGI_HUM1_OLIGO_A_23_P74290 | 0.8064 | **0.0022** |
| IFN regulatory factor 7 | IRF7 | AGI_HUM1_OLIGO_A_24_P118892 | 0.1456 | 0.0596 |
|  | IRF7 | AGI_HUM1_OLIGO_A_24_P378019 | 0.2779 | 0.1135 |
| Phosphodiesterase 4B, cAMP-specific | PDE4B | AGI_HUM1_OLIGO_A_23_P74278 | 0.0897 | 0.6942 |
|  | PDE4B | AGI_HUM1_OLIGO_A_24_P325333 | 0.2021 | 0.2193 |
|  | PDE4B | AGI_HUM1_OLIGO_A_24_P921129 | 0.0389 | 0.6493 |
| TNF alpha-induced protein 6 | TNFAIP6 | AGI_HUM1_OLIGO_A_23_P165624 | -0.0347 | 0.8487 |
| Chemokine (C-C motif) ligand 20 | CCL20 | AGI_HUM1_OLIGO_A_23_P17065 | 0.1011 | 0.5337 |
| Chemokine (C-C motif) ligand 4 | CCL4 | AGI_HUM1_OLIGO_A_23_P207564 | 0.5820 | **0.0031** |
| Chemokine (C-C motif) ligand 5 | CCL5 | AGI_HUM1_OLIGO_A_23_P152838 | 0.3091 | 0.1414 |
| CD69 (p60, early T cell activation antigen) | CD69 | AGI_HUM1_OLIGO_A_23_P87879 | 0.3191 | 0.1978 |
| CD80 (CD28 ligand 1, B7-1) | CD80 | AGI_HUM1_OLIGO_A_23_P155632 | 0.0148 | 0.8539 |
|  | CD80 | AGI_HUM1_OLIGO_A_24_P320033 | 0.1513 | 0.0699 |
| CD86 (CD28 ligand 2, B7-2) | CD86 | AGI_HUM1_OLIGO_A_23_P109988 | 0.4209 | **0.0117** |
|  | CD86 | AGI_HUM1_OLIGO_A_24_P131589 | 0.1260 | 0.7840 |
| Complement factor B (properdin) | CFB | AGI_HUM1_OLIGO_A_23_P156687 | 0.0345 | 0.9284 |
| Chemokine (C-X-C motif) ligand 1 | CXCL1 | AGI_HUM1_OLIGO_A_23_P7144 | 0.4862 | 0.1992 |
| Chemokine (C-X-C motif) ligand 10 | CXCL10 | AGI_HUM1_OLIGO_A_24_P303091 | 0.5991 | **0.0088** |
| Chemokine (C-X-C motif) ligand 11 | CXCL11 | AGI_HUM1_OLIGO_A_23_P125278 | 0.7569 | **0.0204** |
|  | CXCL11 | AGI_HUM1_OLIGO_A_24_P20607 | 0.8111 | **0.0021** |
| Chemokine (C-X-C motif) ligand 9 | CXCL9 | AGI_HUM1_OLIGO_A_23_P18452 | 0.6463 | 0.0534 |
| Fc fragment of IgG, high affinity Ia, receptor (CD64) | FCGR1A | AGI_HUM1_OLIGO_A_23_P63390 | 0.4887 | **0.0269** |
| Fc fragment of IgG, low affinity IIa, receptor (CD32) | FCGR2A | AGI_HUM1_OLIGO_A_24_P927148 | 0.0126 | 0.8439 |
|  | FCGR2A | AGI_HUM1_OLIGO_A_32_P74942 | 0.3426 | **0.0291** |
|  | FCGR2A\|FCGR2C | AGI_HUM1_OLIGO_A_23_P85716 | 0.1737 | 0.1421 |
| Fc fragment of IgG, low affinity IIIa, receptor (CD16a) | FCGR3A | AGI_HUM1_OLIGO_A_23_P200728 | 0.3572 | 0.0727 |
|  | FCGR3A\|FCGR3B | AGI_HUM1_OLIGO_A_23_P126298 | 0.3129 | 0.0619 |
| Inter-cellular adhesion molecule 1 (CD54) | ICAM1 | AGI_HUM1_OLIGO_A_23_P153320 | 0.5131 | **0.0070** |
| Interluekin-12B (NK cell stimulatory factor 2, p40) | IL12B | AGI_HUM1_OLIGO_A_23_P7560 | 0.0534 | 0.4316 |
| Interleukin-18 (IFN-gamma inducing factor) | IL18 | AGI_HUM1_OLIGO_A_23_P104798 | 0.3317 | 0.0648 |
| Interleukin-1 beta | IL1B | AGI_HUM1_OLIGO_A_23_P79518 | 0.2874 | **0.0410** |
| Interleukin-23, alpha subunit p19 | IL23A | AGI_HUM1_OLIGO_A_23_P76078 | 0.1989 | 0.1100 |
| Interleukin-32 | IL32 | AGI_HUM1_OLIGO_A_23_P15146 | 0.3402 | **0.0207** |
| Interleukin-6 | IL6 | AGI_HUM1_OLIGO_A_23_P71037 | 0.0756 | 0.6644 |
| IFN regulatory factor 1 | IRF1 | AGI_HUM1_OLIGO_A_23_P41765 | 0.2917 | **0.0260** |
| Nitric oxide synthase 2A (inducible) | NOS2A | AGI_HUM1_OLIGO_A_23_P502464 | 0.1912 | **0.0330** |
| Suppressor of cytokine signaling 3 | SOCS3 | AGI_HUM1_OLIGO_A_23_P207058 | -0.0348 | 0.8857 |
|  | SOCS3 | AGI_HUM1_OLIGO_A_23_P351069 | 0.0053 | 0.9442 |
| Toll-like receptor 2 | TLR2 | AGI_HUM1_OLIGO_A_23_P92499 | 0.4668 | **0.0082** |
| Toll-like receptor 4 | TLR4 | AGI_HUM1_OLIGO_A_23_P60306 | 0.2192 | 0.1173 |
|  | TLR4 | AGI_HUM1_OLIGO_A_24_P69538 | 0.0796 | 0.6752 |
|  | TLR4 | AGI_HUM1_OLIGO_A_32_P66881 | 0.2253 | 0.2071 |
| Tumor necrosis factor | TNF | AGI_HUM1_OLIGO_A_23_P376488 | 0.3645 | **0.0213** |
|  | TNF | AGI_HUM1_OLIGO_A_24_P50759 | 0.0670 | 0.3157 |
| TNF (ligand) super family member 10 | TNFSF10 | AGI_HUM1_OLIGO_A_23_P121253 | -0.1911 | 0.4498 |
|  | TNFSF10 | AGI_HUM1_OLIGO_A_32_P126023 | 0.1063 | 0.3628 |

**M2 GENES**

| Gene name | Symbol | Probe set ID | difference^a^ | p value^b^ |
| --- | --- | --- | --- | --- |
| Adenosine A3 receptor | ADORA3 | AGI_HUM1_OLIGO_A_23_P126540 | 0.0533 | 0.5474 |
|  | ADORA3 | AGI_HUM1_OLIGO_A_23_P137931 | 0.1294 | 0.3291 |
| Arginase | ARG1 | AGI_HUM1_OLIGO_A_23_P111321 | 0.0676 | 0.5560 |
| Collagen, type VI, alpha 2 | COL6A2 | AGI_HUM1_OLIGO_A_23_P211233 | 0.0907 | 0.6824 |
|  | COL6A2 | AGI_HUM1_OLIGO_A_23_P310956 | 0.0179 | 0.9178 |
|  | COL6A2 | AGI_HUM1_OLIGO_A_32_P22750 | 0.0905 | 0.5344 |
| Growth arrest-specific 7 | GAS7 | AGI_HUM1_OLIGO_A_24_P395610 | 0.1593 | 0.2454 |
|  | GAS7 | AGI_HUM1_OLIGO_A_24_P82466 | 0.0609 | 0.6386 |
| Heparan sulfate (glucosamine) 3-O-sulfotransferase 1 | HS3ST1 | AGI_HUM1_OLIGO_A_23_P121657 | 0.0658 | 0.7617 |
| c-mer proto-oncogene tyrosine kinase | MERTK | AGI_HUM1_OLIGO_A_23_P32955 | 0.2471 | 0.0789 |
| Purinergic receptor P2Y, G protein-coupled 5 | P2RY5 | AGI_HUM1_OLIGO_A_23_P2705 | -0.2867 | 0.0576 |
| Stabilin 1 | STAB1 | AGI_HUM1_OLIGO_A_23_P32500 | -0.1101 | 0.4543 |
|  | STAB1 | AGI_HUM1_OLIGO_A_24_P119456 | -0.0014 | 0.9862 |
| Chemokine (C-C motif) ligand 17 | CCL17 | AGI_HUM1_OLIGO_A_23_P26325 | 0.1346 | 0.4808 |
| Chemokine (C-C motif) ligand 18 | CCL18 | AGI_HUM1_OLIGO_A_23_P55270 | 0.5021 | **0.0283** |
| Chemokine (C-C motif) ligand 23 | CCL23 | AGI_HUM1_OLIGO_A_24_P133905 | -0.1500 | 0.2903 |
|  | CCL23 | AGI_HUM1_OLIGO_A_24_P319088 | -0.0090 | 0.9338 |
| Chemokine (C-C motif) ligand 24 | CCL24 | AGI_HUM1_OLIGO_A_23_P215491 | -0.0894 | 0.4356 |
| CD163, scavenger receptor | CD163 | AGI_HUM1_OLIGO_A_23_P33723 | 0.4804 | 0.0531 |
| CD36, collagen type I receptor | CD36 | AGI_HUM1_OLIGO_A_23_P111583 | -0.6550 | **0.0347** |
|  | CD36 | AGI_HUM1_OLIGO_A_24_P925505 | -0.4578 | **0.0438** |
| CD9, tetraspanin | CD9 | AGI_HUM1_OLIGO_A_23_P76364 | -0.3266 | 0.2346 |
| C-type lectin domain family 7A | CLEC7A | AGI_HUM1_OLIGO_A_23_P2640 | 0.1326 | 0.3077 |
|  | CLEC7A | AGI_HUM1_OLIGO_A_24_P227415 | -0.0074 | 0.9431 |
| Chemokine (C-X-C motif) receptor 4 | CXCR4 | AGI_HUM1_OLIGO_A_23_P102000 | 0.0641 | 0.7902 |
| Fc fragment of IgE, low affinity II, receptor (CD23) | FCER2 | AGI_HUM1_OLIGO_A_23_P164773 | 0.0490 | 0.6455 |
| Fibronectin | FN1 | AGI_HUM1_OLIGO_A_24_P119745 | 0.0164 | 0.9557 |
|  | FN1 | AGI_HUM1_OLIGO_A_24_P334130 | 0.1934 | 0.2602 |
|  | FN1 | AGI_HUM1_OLIGO_A_24_P85539 | 0.1223 | 0.6293 |
|  | FN1 | AGI_HUM1_OLIGO_A_32_P201723 | 0.1437 | 0.4195 |
|  | FN1 | AGI_HUM1_OLIGO_A_32_P92642 | 0.0994 | 0.6351 |
| Interleukin-10 | IL10 | AGI_HUM1_OLIGO_A_23_P126735 | 0.0740 | 0.5792 |
| Interleukin-1 receptor antagonist | IL1RN | AGI_HUM1_OLIGO_A_23_P209995 | 0.0176 | 0.9236 |
|  | IL1RN | AGI_HUM1_OLIGO_A_32_P71710 | 0.0761 | 0.5273 |
| Interleukin-4 receptor | IL4R | AGI_HUM1_OLIGO_A_23_P129556 | 0.0953 | 0.5081 |
| Matrix metalloproteinase-2 (gelatinase A) | MMP2 | AGI_HUM1_OLIGO_A_32_P35906 | 0.1942 | 0.1149 |
| Matrix metalloproteinase-7 (matrilysin) | MMP7 | AGI_HUM1_OLIGO_A_23_P52761 | 0.5024 | 0.1361 |
| Matrix metalloproteinase-9 (gelatinase B) | MMP9 | AGI_HUM1_OLIGO_A_23_P40174 | 0.4144 | 0.1832 |
| Mannose receptor C type 1 | MRC1 | AGI_HUM1_OLIGO_A_23_P12746 | 0.2056 | 0.1612 |
| Mannose receptor C type 2 | MRC2 | AGI_HUM1_OLIGO_A_23_P49627 | -0.0097 | 0.9294 |
| Macrophage scavenger receptor 1 | MSR1 | AGI_HUM1_OLIGO_A_23_P216176 | 0.0713 | 0.6701 |
|  | MSR1 | AGI_HUM1_OLIGO_A_23_P392942 | 0.1344 | 0.2974 |
|  | MSR1 | AGI_HUM1_OLIGO_A_24_P372223 | 0.2359 | 0.3044 |
| Transforming growth factor beta 1 | TGFB1 | AGI_HUM1_OLIGO_A_24_P79054 | 0.0626 | 0.6676 |

a. difference of mean expression values (high PCNA^+^ TAMs - low PCNA^+^ TAMs)

b. Student’s t-test; significant differences (p<0.05) shown in bold
