# Supplementary material for: The Prognostic Implications of Macrophages Expressing Proliferating Cell Nuclear Antigen in Breast Cancer Depend on Immune Context
Source: PLoS One. 2013 Oct 29;8(10):e79114. doi: 10.1371/journal.pone.0079114 (PMC3812150; doi:10.1371/journal.pone.0079114)
Supplement: Table S2 — Association of M1- and M2-related differential gene expression with clinical parameters in breast cancer. (DOCX) [file pone.0079114.s003.docx]

**Table S2. Association of M1- and M2-related differential gene expression with clinical parameters in breast cancer.**

**M1 GENES**

| Gene | High PCNA+ TAMs vs low PCNA+ TAMs | HR-pos vs HR-neg | Low grade vs high grade | RCB 3 vs RCB 0/1 | Recurrence vs recurrence-free |
| --- | --- | --- | --- | --- | --- |
| CCL4 | 0.582 (0.003) |  |  | 0.527 (0.032) | 0.422 (0.046) |
| CCL5 |  |  |  | 0.761 (0.010) |  |
| CD69 |  |  |  | 0.806 (0.017) |  |
| CD80 |  | 0.166 (0.048) | 0.193 (0.020) |  |  |
| CD86 | 0.421 (0.012) |  | 0.371 (0.027) | 0.493 (0.030) | 0.402 (0.024) |
| CFB |  | -1.361 (0.0003) | -0.872 (0.021) |  |  |
| CXCL1 |  | 0.991 (0.008) | 1.072 (0.004) |  |  |
| CXCL10 | 0.599 (0.009) |  |  |  |  |
| CXCL11 | 0.811 (0.002) |  | 0.609 (0.022) |  |  |
| CXCL9 |  |  |  |  | 0.789 (0.026) |
| FCGR1 | 0.489 (0.027) |  |  |  |  |
| FCGR2A | 0.343 (0.029) |  |  |  |  |
| FCGR3A |  | 0.364 (0.030) |  |  |  |
| GBP1 |  | 0.732 (0.015) |  |  |  |
| GBP2 |  |  |  | 0.487 (0.036) | 0.531 (0.014) |
| GBP3 |  |  |  |  | 0.715 (0.006) |
| GBP4 | 0.634 (0.019) |  |  | 0.853 (0.007) | 0.613 (0.034) |
| GBP5 | 0.806 (0.002) |  | 0.586 (0.028) |  |  |
| ICAM1 | 0.513 (0.007) | 0.401 (0.037) | 0.543 (0.004) |  |  |
| IL18 |  | 0.535 (0.003) |  |  |  |
| IL1B | 0.287 (0.041) |  |  |  |  |
| IL32 | 0.340 (0.021) | 0.312 (0.035) |  | 0.429 (0.048) |  |
| IL6 |  | 0.413 (0.017) |  |  |  |
| IRF1 | 0.292 (0.026) |  |  |  | 0.284 (0.041) |
| IRF7 |  | 0.162 (0.036) |  |  |  |
| NOS2A | 0.191 (0.033) |  |  |  |  |
| PDE4B |  | 0.435 (0.008) |  |  |  |
| TLR2 | 0.467 (0.008) |  | 0.545 (0.002) |  |  |
| TNF | 0.365 (0.021) | 0.161 (0.015) |  |  |  |
| TNFAIP6 |  |  |  |  | 0.387 (0.043) |
| TNFSF10 |  |  |  |  | 0.600 (0.024) |

**M2 GENES**

| Gene | High PCNA+ TAMs vs low PCNA+ TAMs | HR-pos vs HR-neg | Low grade vs high grade | RCB 3 vs RCB 0/1 | Recurrence vs recurrence-free |
| --- | --- | --- | --- | --- | --- |
| CCL18 | 0.502 (0.029) |  | 0.860 (0.0001) | 0.648 (0.042) |  |
| CD36 | -0.655 (0.035) |  |  |  |  |
| CLEC7A |  |  |  | 0.426 (0.022) | 0.296 (0.031) |
| COL6A2 |  |  |  |  | 0.473 (0.043) |
| CXCR4 |  |  |  |  | 0.511 (0.044) |
| FN1 |  | 0.384 (0.030) |  |  |  |
| GAS7 |  |  |  |  | 0.290 (0.033) |
| HS3ST1 |  | 0.519 (0.016) |  |  |  |
| IL1RN |  |  | 0.470 (0.009) |  |  |
| IL4R |  |  |  |  | 0.354 (0.019) |
| MERTK |  |  | 0.359 (0.010) |  |  |
| MMP7 |  | 0.789 (0.019) |  |  |  |
| MMP9 |  |  |  | 0.892 (0.044) | 0.858 (0.009) |
| MSR1 |  |  |  |  | 0.270 (0.047) |
| P2RYS |  |  | -0.446 (0.003) |  | 0.322 (0.044) |

The difference of mean expression values (high PCNA^+^ TAMs minus low PCNA^+^ TAMs, HR-neg minus HR-pos; high grade minus low grade; RCB 0/1 minus RCB 3; Recurrence-free minus recurrence) are listed only for those genes that displayed differential expression. Student’s t-test p values are given in parentheses. These values are not corrected for multiple comparisons.
